# A plea for using qualitative aspects in the interpretation of ecological field data as revealed by the proof of carabid beetle assemblages of a pristine salt marsh

**DOI:** 10.3897/zookeys.100.1532

**Published:** 2011-05-20

**Authors:** Dietrich Mossakowski, Wolfgang Dormann

**Affiliations:** 1 Seeweg 10, D-23942 Groß Schwansee; 2 Am Rolandsgraben 11, D-28865 Lilienthal

**Keywords:** Cluster analysis, indicator value, qualitative interpretation, ecological field data, Carabidae

## Abstract

The evaluation of ecological field data can be done by an increasing number of quantitative methods. The application of these methods often is often blind against two kinds of problems: (i) the data often do not meet the requirements of a method, e.g., as an ultra-metric structure of the data in the case of hierarchical cluster analysis. In such cases, the result will be misleading because the presentation of results is ultra-metric independent on the structure of the data. (ii) Most of the animals are able to move actively or may drift passively by wind, etc. Therefore, species occurring by accident like vagrants have to be eliminated from the assemblage of animals at a particular site before a quantitative method is applied. In addition, the result of a quantitative analysis has to be checked for its ecological plausibility. This is a qualitative step, which can only be done by taking into account the known data on biology and ecology of the species.

Some pitfalls of an exclusive application of quantitative methods will be demonstrated in this paper using a data set of salt marsh Carabidae.

## Introduction

In the literature, the representation and evaluation of ecological field data is achieved by a broad spectrum of different methods ranging from vegetation tables to trees of similarity of sites. McGeoch (1998) recommended a nine-step procedure for the evaluation of ecological field data in a paper on terrestrial insects as bioindicators. She explicitly demands that ecologists use quantitative data and procedures including statistics. In general, quantitative data are indispensable, and the interpretation and representation of data by quantitative methods is a must. But two kinds of problems exist when a quantitative method is applied blindly. (i) The data often do not meet the requirements of a method, e.g., as an ultra-metric structure of the data in the case of hierarchical cluster analysis. In such cases, the result will be misleading because the presentation of results is ultra-metric independent on the structure of the data. (ii) Most of the animals are able to move actively or may drift passively by wind etc. Therefore, species occurring by accident like vagrants have to be eliminated from the assemblage of animals at a particular site before a quantitative method is applied. In addition, the result of a quantitative analysis has to be checked for its ecological plausibility. This is a qualitative step, which can only be done by taking into account the known data on biology and ecology of the species.

Dufrène and Legendre (1997) developed the ‘Indicator Value’ (IndVal) method, which combines data on both abundance and frequency in an optimal manner. McGeoch and Chown (1998) published an enthusiastic review of the IndVal method entitled “Scaling up the value of bioindicators”. Subsequently, this method was applied in many studies. In the intervening period, this method has been extended by Clarke et al. (2006; zero-adjusted Bray–Curtis coefficient), Dai et al. (2006; Total Indicator Value Method), and Bakker (2008; improvement of permutation test, consistency of index and binary data).

We use mainly the IndVal method to call attention to some problems of the application of quantitative methods and to show that qualitative aspects have to be included for data interpretation. In this paper the following questions are addressed:

§ Hierarchical cluster analyses were often used to generate trees to arrange sites by the similarity of their faunal assemblages. What are the objectionable effects of these methods?

§ Is the IndVal index simple and based only on within-species abundance and occurrence comparisons, without any comparison among species?

§ Is it wise to always use the maximum of IndVal?

§ What is the impact of a qualitative approach?

Salt marshes are considered to be optimal for the purpose of this paper because they offer a structured elevation gradient and they are an extreme habitat for carabid beetles (Mossakowski 2007): a low number of stenotopic species occur in high abundances in particular in lower salt marsh zones.

## Material and Methods

### Material

The test data were collected in a project on salt marshes and climate impact (Dormann et al. 2000, Dormann et al. 2008) on the pristine salt marshes of the East Frisian island Mellum, Germany. Pitfall traps were exposed during the seasons of three years from April to October in different configurations. To avoid damage from the tide and waves, an air-bell trap (Dormann 2000) was constructed and exposed at the lower salt marsh sites, between 20 cm below Mean High Water Level (MHW) (-20), at MHW and up to 40 cm above MHW. Only ‘year’ catches of 1998 (April to October) were used and numbered by elevation (Table 1). At 100 cm above MHW, three sites were selected due to different soil conditions at this elevation and indicated by adding an integer to the last position of the site number (101, 102, 103). Five traps were exposed at each site. Each trap was numbered with site elevation and a digit (–20-5: trap five at site –20; 101-1: trap one at site one of elevation 100).

**Table 1.** Elevation gradient and number of exposed pitfall traps in the salt marshes of Mellum.

		↓	Elevation above MHV (cm)											
-20		0	10	20		40		60		80		100		120
Number of pitfall traps														
5		5	5	5		5		5		5		3×5		5

### Quantitative methods

1. IndVal of Dufrène and Legendre

In our opinion, the Indicator Value (IndVal) method of Dufrène and Legendre (1997) comprises three steps: (i) the arrangement of catches/sites. The data are represented in a tree constructed preferably from the distances in the species-site matrix. Dufrène and Legendre (1997) use a non-hierarchical cluster analysis in their paper but in their original program a hierarchical one is required. (ii) The information of the resulting tree must be transformed by hand into a matrix, which reflects hierarchically the arrangement of sites in the tree. (iii) The appropriate IndVal search for characteristic species: The maximum IndVal is calculated using the fidelity and specificity of a species for groups of sites that are taken from the tree via the matrix of step ii.

Test calculations with our data were performed with the original IndVal program (IndVal 2.0; Dufréne & Legendre 1997) using Ward’s method with Relative Euclidean distances as well as with UPGMA with Bray-Curtis (Sœrensen) distances (step i). The problems of hierarchical cluster analysis were demonstrated by a calculation with the full data set. A recalculation was done after deletion of two sites (102 and 103).

The impact of the tree structure (generated in step ii) on the result (step iii) is shown by a comparison of a hierarchical tree with a freehand produced tree on the basis of the site specific data.

2. The IndVal procedure of PC-ORD (McCune and Mefford 2006) was applied with the same data and a series of free-hand produced trees.

3. Other quantitative methods are applied to the identical sets of data: Principal Coordinate Analysis (PCO)/MVSP; Discriminant Analysis/Brodgar; Multivariate Partitioning (mvpart) Brodgar/R.

Applied statistics for IndVal: Random permutation test (999). Significance level: 0.01.

### Qualitative methods

A table of year-catches for species x traps is presented (Appendix III), which covers the original year-catch numbers in an arrangement like that in vegetation tables. These data were freehand interpreted under consideration of the specific conditions at the study sites and the biological and ecological demands of the species.

## Results

### Quantitative evaluation: IndVal original program

**Figure 1.**
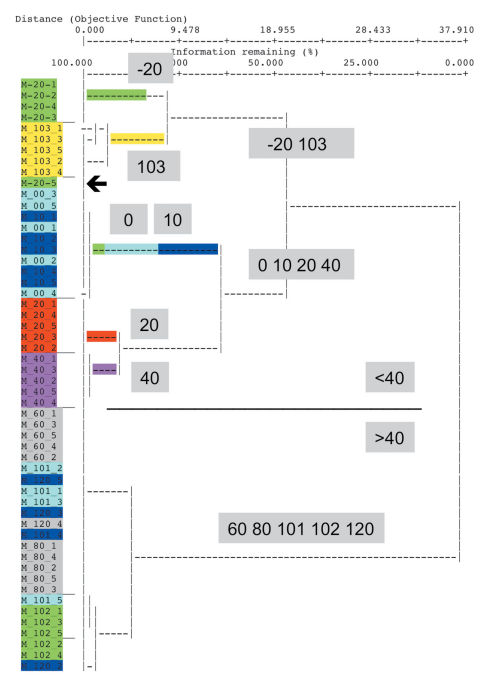
Result of a cluster analysis using Relative Euclidean distances and Ward’s method. Most traps of the site at the lowest elevation (-20 cm below MHW) cluster with those of 100 cm above MHW. Arrow: One trap of -20 behaves differently.

The first step of the IndVal procedure yielded similar results with different procedures. In order to demonstrate characteristic effects of cluster methods, the result of Ward’s method with Relative Euclidean distances including all trap-sites is presented in Fig. 1. In the resulting tree, two sites of very different elevation levels clustered together: four out of five traps of site –20 and all traps of site 103. They were placed together with another cluster of 0, 10, 20, 40 and the fifth trap of –20 (–20-5). All remaining sites of higher elevation (60–120) clustered closely together.

The result obtained by UPGMA with Bray-Curtis distances also showed a basic split of –20 against the cluster of 0, 10, 20, 40 and –20-5. At the other end, 103 splits off at the basis of all the sites at higher elevation.

Elimination of site 102 and 103 resulted in more plausible trees. In the case of Ward’s method with Relative Euclidean distances, the traps of elevation –20 and those of site 20 and 40 were put in the cluster next to that of 0 and 10, which included trap –20-5.

In the second step of the original IndVal procedure, the information of the tree was transformed into a hierarchical notification (Appendix I). In order to get a clearly arranged result, the tree of the first step was simplified, as was the matrix for the calculation of the IndVal values. Sites 103 and 102 were omitted and all five traps of equal elevation were assigned to the same group.

**Figure 2.**
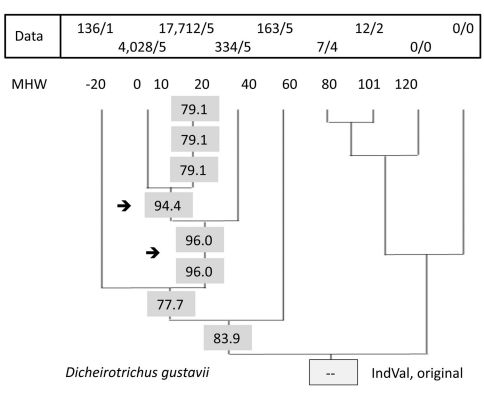
IndVals at different levels in the UPGMA tree. Result for a single species, *Dicheirotrichus gustavii*, calculated by the original IndVal program. Eight values of the nine levels are significant. Data: abundance/frequency data. 7/4: a total of seven specimens were found in four of the five traps. Sites 102 and 103 are omitted.

The third step was performed first by the original IndVal program. Fig. 2 demonstrates the distribution of successive IndVal’s at different levels of this simplified tree showing the result of one calculation for a single species. As an example, *Dicheirotrichus gustavii* was chosen as a highly abundant and specific species in salt marshes. All values shown are significant.

**Figure 3.**
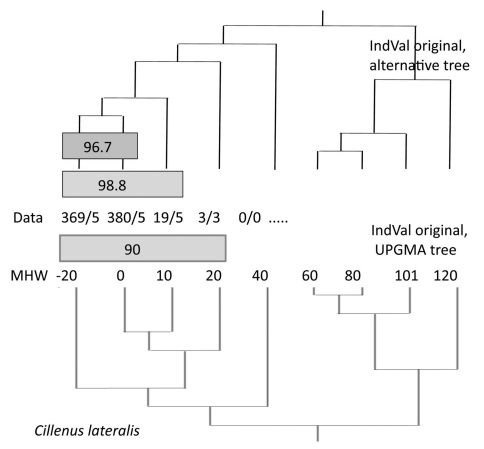
Results of the IndVal procedure depend on the tree used. Data: abundance/frequency of *Cillenus lateralis* along the elevation gradient. 3/3: a total of three specimens was found in three of the five traps. Sites 102 and 103 are omitted.

A result for *Cillenus lateralis* is shown in Fig. 3 in order to show the dependence of the IndVals on the tree structure. In the lower section, the original (simplified) matrix was used. A maximum indicator value of 90% was found for this species (sites –20 to 20) by the original IndVal program. In a calculation using a free-hand self-constructed, alternative tree, higher values were found.

### Quantitative evaluation: IndVal by PC-ORD

The application of the same data to the IndVal procedure of PC-ORD was carried out by different arrangements of sites to groups. The obvious first step was to take the five traps per elevation as groups (first data line in Table 2). Each result consists of a table of IndVals in which scores for each species and the chosen arrangement of groups are listed. As an example of differing results from multiple calculations with changing arrangements, the scores for *Cillenus lateralis* are listed in Table 2. The notation results differ from those of the original IndVal program; scores were not listed hierarchically, they were listed parallel. Therefore, scores of other groups apart from the maximum can be evaluated.

**Table 2.** Indicator Values as a result of multiple calculations performed by PC-ORD. Results for *Cillenus lateralis*. Each line represents a separate calculation with the groups indicated by vertical lines. Bold face numbers indicate significance. **MHW**: Mean High Water Level.

*MHV*	*-20*		*0*		*10*		*20*		*40*		*60*		*80*		*101*		*120*
(1)	48	|		|		|		|		|		|		|		|	
(2)		*100*		|						|							
(3)	*65*	|		34		|				|							
(4)		*98*		|		1		|				|				|	
(5)		*98*		|		1		|		|				|			
(6)	*85*	|				7				|							

### Quantitative methods: Principal Coordinate Analysis

The test data were also applied to methods that do not use distance-based algorithms. As an example, the result of Principal Coordinate Analysis (PCO) is shown in the Appendix II. Similar to the grouping by distance methods, the lower sites form one group while the higher sites form another. One trap at site –20 is also positioned close to those of higher elevation (site 0, 10 etc). The position of site 103 is remarkable because it is arranged near to the site with the lowest elevation – far from those at equal elevation (101 and 102). The results of Discriminant Analysis and Multivariate Partitioning (not shown here) display similar effects.

### Qualitative methods

In order to apply qualitative aspects of interpretation, we present the full data set in the Appendix III in order to enable the reader to evaluate our statements. We focus on two species, characteristic in a different way, of flooded and salty habitats.

The occurrence of *Dicheirotrichus gustavii* along the elevation gradient is characterised by very high numbers at an elevation 10 cm above MHW (site 10 with a mean of 3606, range 1181 - 5844 specimens per year-trap) and at MHW (site 0 with a mean of 824, range 256 - 1355). This species was found only in one trap at lower elevation (–20-5) and in moderate numbers at higher sites.

*Cillenus lateralis* was collected in traps of elevation –20 and 0 in moderate numbers (–20: mean = 74, range: 6–309; 0: mean = 76, range 6–161). This species occurred with single specimens at elevations 10 and 20.

Habitat conditions at the lower part of the Mellum salt marshes differ markedly in soil and flood frequency: sites –20 and 0 contain about 70–80% sand, they flooded regularly, at least once per day (1 – 1.5 times per day). Above this level, 10 to 80 cm above MHW, the soil consists of a high amount of clay and a low sand content. Site 10 is flooded about 0.7 times, site 20 about 0.5 times, and site 40 lower than 0.2 times per day.

## Discussion

### Effects of cluster methods

There are two unexpected results in Fig. 1: (i) the position of trap –20-5 and (ii) that of site 103. These effects do occur also in results of multivariate methods but shall be discussed using the example of cluster analysis.

In particular, the positioning effects mentioned afore can be interpreted by viewing the data in detail. (i) In trap –20-5, *Dicheirotrichus gustavii* was caught but is missing completely in the other traps at elevation –20. But the number of this species found in this deviant trap is very low in comparison with the very high abundance at higher elevations (see full data in Appendix III). We have to take into account that these specimens are migrants from higher sites. (ii) The position of all the traps of site 103 depends on quite a different assemblage of species, which is obviously different not only from those at comparable elevations but also from all sites. This depends on the differences in sand content and wetness between the three sites at 100 cm above MHW: site 103 is a very dry, sandy habitat, and consequently, the assemblage of species is quite different (Table 3).

**Table 3.** Selected carabid species to show differences at site 100 (100 cm above MHW). Only species with characteristic distribution (more or less exclusive or missing) are included. The catches of five traps per site are summarized.

*Elevation Taxon*	*-20*	*0*	*10*	*20*	*40*	*60*	*80*	*101*	*102*	*103*	*120*
*Calathus erratus*			2							89	1
*Amara fulva*										30	
*Amara spreta*										20	
*Calathus ochropterus*										19	
*Harpalus affinis*										2	
*Trechoblemus micros*										1	
*Dicheirotrichus gustavii*	136	4134	18687	334	163	7	12			7	
*Bembidion minimum*			2		761	4				2	
*Bembidion guttula*								1	1		
*Badister bullatus*								14	1		17
*Badister sodalis*							4	22			10
*Pterostichus niger*						13	280	264	168		130
*Calathus fuscipes*		3	2		2	37	59	216	164	1	368
*Dyschirius globosus*	1		1		6	562	1210	1030	267	2	923

Because the clustering process will put the tho step most similar sites together in a step-by-step approach, site 103 and most traps of –20 remain at the end of the clustering process.

In general, a basic problem of distance methods is that trees showing similarity of sites are the result of a cluster analysis. This is critical because the condition for use, the existence of metric or ultra-metric data (Appendix IV), is often not realized in ecological field data and neither tested nor discussed by many authors. In the example demonstrated, this problem is easy to see. But the problem exists also in the case of data with a structure closer to an ultra-metric one. But it will not be as obvious as in our example. The distance matrix can be tested for ultra-metric conditions by checking each triplicate of values whether the strengthened triangle inequality is given (see Appendix IV). But the programs do not output the distance matrix.

### Transformation from tree to matrix

In the original IndVal program, a hierarchical tree is specified. Arranging a matrix with the correct information requires some patience. This may be because it is done for the first time, or because trees are usually being used for phylogenies. As such, this procedure is not simple.

### IndVal based only on within-species data

This statement is only correct when considering the last step of IndVal evaluation. However, as has already been stated by Dufrène and Legendre (1997), and shown in Fig. 3, the resulting IndVal of a species depends on the arrangement of sites to groups, the corresponding tree or matrix. Therefore, the data of the total assemblage have an indirect influence on the IndVal scores. This is true, not only when using a cluster analysis but also for other techniques.

### Qualitative interpretation and IndVal maximum

As an example, the data and IndVals for *Dicheirotrichus gustavii* are shown in Fig. 2. The highest value for this species (96%) was found for a group of sites; 0, 10, and 20. However, sites 0 and 10 form a group with an index (94%) similar to the former. Both are significant. But what is the difference? How can it be tested? Compared to the data for sites 0 and 10, the relatively low numbers below and above this elevation may indicate a suboptimal habitat for this species. Otherwise, we have to take into account that these beetles are able to walk and to fly or they may drift during flooding, which occurs at least once per day at this elevation. Thus, we prefer to take this species as an indicator for sites at elevation 0 and 10 (see also Appendix III).

The same problem can be identified for *Cillenus lateralis* (Fig. 3). A purely quantitative view will find an IndVal of max. 98.8%. But if we consider qualitative data, our knowledge of the ecology and biology of the species, the lower value (96.7%; for sites 0 and –20) is the appropriate one. *Cillenus lateralis* inhabits more or less pure sandy soils (about 70–80% sand at site –20 and 0 on Mellum), which must be flooded regularly. These conditions are only realized at this elevation. Specimens occurring above this level have to be classified as vagrants.

## Conclusions

1. The construction of a tree using distance data by hierarchical cluster analysis always results in an ultra-metric tree although the data are not ultra-metric. Therefore, such procedures should not be used. Also non-distance methods yielded problematic results with the data set under study.

2. Because the original IndVal program requires a hierarchical tree transformed into a matrix, which is also structured hierarchically, we recommend using the IndVal function in PC-ORD as a simple procedure (not free of charge). The Mac version of the original IndVal program does not run on IntelMac. See also Bakker (2008; appendix: program in R).

3. The IndVal method is not only based on the within-species data because the arrangement of sites to groups depends on the whole data set.

4. The examples of *Cillenus* and *Dicheirotrichus* demonstrate that a quantitative analysis may involve some pitfall traps, e.g. the maximum of IndVal. An additional qualitative interpretation is necessary which incorporates biological and ecological data known for the species. It has to be remembered that a particular study never represents more than a small sample of the complete diversity. Thus, external data should be incorporated in order to avoid a narrow focus on one’s own limited set of data. Large and good data sets on species and sites are presented by our colleagues in the Netherlands (Alders et al. 1991, Turin 2000).

5. The necessity to incorporate qualitative aspects is also an argument against the use of only binary (presence/absence) data recently proposed by Bakker (2008).

6. The classic characterization of ecological field data along habitat preference classes should be revived. As a student, D.M. learned from Wolfgang Tischler (1949) that we have to eliminate non-indigenous species such as vagrants - even if they occur in larger numbers.

Consequently, a more qualitative evaluation requires the publication of a detailed specification of methods and of species x site data as done or requested by Dufrène and Legendre (1997), Desender et al. (2007) and Bakker (2008).

## Appendix I

Arrangement of sites represented by a tree and a matrix. (doi: 10.3897/zookeys.100.1532.app1) File format: Adobe Arcobat PDF.

**Explanation note:** Sites are numbered by elevation (right column). The structure of the tree is displayed by a hierarchical notification of the matrix.

Copyright notice: This dataset is made available under the Open Database License (http://opendatacommons.org/licenses/odbl/1.0/). The Open Database License (ODbL) is a license agreement intended to allow users to freely share, modify, and use this Dataset while maintaining this same freedom for others, provided that the original source and author(s) are credited.

## Appendix II

Principal Coordinate Analysis of the whole data set. (doi: 10.3897/zookeys.100.1532.app2) File format: Adobe Arcobat PDF.

Copyright notice: This dataset is made available under the Open Database License (http://opendatacommons.org/licenses/odbl/1.0/). The Open Database License (ODbL) is a license agreement intended to allow users to freely share, modify, and use this Dataset while maintaining this same freedom for others, provided that the original source and author(s) are credited.

## Appendix III

Catches of carabid beetles on the island of Mellum. (doi: 10.3897/zookeys.100.1532.app3) File format: HTML.

**Explanation note:** Yellow colour indicates congruence between a pure quantitative and our qualitative interpretation. Blue colour indicates additional significant IndVal's due to pure quantitative results. Halobiontic and halophilic species are listed in the upper section (above first break). The first 30 species (above second break) are used for the calculations of IndVals. Sites were numbered by their elevation above MHW [cm]. 1,2...5: trap

Copyright notice: This dataset is made available under the Open Database License (http://opendatacommons.org/licenses/odbl/1.0/). The Open Database License (ODbL) is a license agreement intended to allow users to freely share, modify, and use this Dataset while maintaining this same freedom for others, provided that the original source and author(s) are credited.

## Appendix IV

Triangles and trees, the distance matrices of which fulfil metric or ultra‐metric conditions respectively. (doi: 10.3897/zookeys.100.1532.app4) File format: Adobe Arcobat PDF.

Copyright notice: This dataset is made available under the Open Database License (http://opendatacommons.org/licenses/odbl/1.0/). The Open Database License (ODbL) is a license agreement intended to allow users to freely share, modify, and use this Dataset while maintaining this same freedom for others, provided that the original source and author(s) are credited.
